# Sensitivity analysis of heat and mass transfer at working face in high-temperature mine

**DOI:** 10.1371/journal.pone.0306269

**Published:** 2024-06-28

**Authors:** Hang Zhou, Xiangdong Zhang, Shuguang Zhang

**Affiliations:** 1 School of Civil Engineering, Liaoning Technical University, Fuxin, China; 2 Guangxi Key Laboratory of Geotechnical Mechanics and Engineering, Guilin University of Technology, Guilin, China; Tongji University, CHINA

## Abstract

Thermal damage from heat sources severely affects the safety of deep mine production. Heat and mass transfer between heat sources and airflow leads to the increase of the airflow temperature (AFT), moisture content of airflow (AFMC) and relative humidity of airflow (AFRH). This study aims to quantify uncertainty contributions of the working face parameters on AFT, AFMC and AFRH and find their main contributors. The flow, geometric and physical parameters are chosen as uncertainty sources. Subsequently, Sobol indices are obtained using the point-collocation non-intrusive polynomial chaos method, denoting the sensitivity of each input parameter. It was found that the inflow wind temperature and the wind velocity are two top factors influencing AFT and AFMC, while relative humidity of inflow wind and the wind velocity are two top factors influencing AFRH. In the single factor analysis, the uncertainty contributions of the inflow wind temperature on AFT and AFMC, and relative humidity of inflow wind on AFRH can exceed 0.7, which is higher than those of the wind velocity. The geometric parameters of the working face, namely the length, width and height, and ventilation time are also significant quantities influencing AFT, AFMC and AFRH. Compared to AFT and AFMC, two other significant quantities influencing AFRH are the thermal conductivity of coal and the original temperature of the rock.

## 1. Introduction

High-temperature thermal damage severely affects the safety of coal mine production. The effective measures must thus be implemented to control the thermal damage [1–3]. The mechanical cooling technology has been widely used in high-temperature mines [4–6]. The cooling load of the working face is an important parameter for optimizing the cooling system, which is crucial to achieve the cooling effect [7]. The cooling load can be calculated by the analysis method of heat sources, the simplified method and the back-analysis method [8].

The heat and mass transfer process between the surrounding rock and airflow is a complex engineering problem. The amounts of sensible heat and latent heat are the basis for calculating the airflow temperature (AFT), moisture content of airflow (AFMC) and relative humidity of airflow (AFRH) [9]. Li et al. [10] analyzed the heat transfer between the surrounding rock and airflow according to the dynamic grid method, and obtained the AFT change when an auxiliary ventilation system was adopted. Yi et al. [11] analyzed the dynamic mechanism of tunnel AFT with the seasonal variation by the measured temperature distribution. Zhang et al. [12] proposed a simulated model of the heat transfer at three-dimensional scale based on the radiation cooling mechanism, and analyzed the influence of the inflow parameters on AFT. Moreover, it was found that the stratified rock can lead to the significant difference in the temperature field, compared with the homogeneous assumption [13].

The AFRH of the working face can range from 90% to 100% due to abundant water in coal mines [14, 15], which severely endangers the physical and mental health of the workers. Luo [16] and Luis [17] et al. analyzed tunnel AFRH by experimental and numerical methods, respectively. Zhang et al. [18] established the evaporative cooling system and investigated the cooling effect under different water-sprayed conditions. Chen et al. [19] proposed mine ventilation network of the air-volume coupling iteration based on differential equations of heat and mass transfer. Previous studies show that inflow parameters determine the cooling effect of ventilation [20, 21]. Hence, sensitivity analyses of the inflow parameters to AFT and AFRH are necessary to find their main contributors.

Many techniques and methodologies for sensitivity analyses have been proposed [22, 23]. The variance analysis method is normally utilized for work optimization [24, 25]. The Monte Carlo method commonly requires a substantial number of samples, thus leading to high run costs [26]. In recent studies, surrogate models building an exact relation between model inputs and responses are given sufficient attention, including the Gaussian process regression method [27], the Bayesian model averaging method [28] and polynomial chaos expansion methods [29]. Sobol indices proposed based on the variance decomposition theory can be calculated by using the polynomial chaos expansion coefficients [30, 31]. Therefore, polynomial chaos expansion methods are widely utilized to analyze sensitivities in various engineering problems, such as aerodynamic heating [32], fluid flow [33] and multiscale methods [34], owing to their solid feasibility and mathematical foundation. The intrusive polynomial chaos method still needs a deterministic relationship between inputs and responses [35], requiring revised programs in CFD solvers. However, the non-intrusive polynomial chaos (NIPC) method does not resolve the exact relationship between input parameters and responses, instead bridges this relationship using CFD solvers considered as a black box [36, 37], thus enhancing the usability of the NIPC method. Hosder et al. [38] applied the point-collocation NIPC technique to propagate the uncertainties in CFD simulations and proved the NIPC method efficiency. Wang et al. [39] employed the point-collocation NIPC method to investigate the uncertainties of geometric parameters in heat transfer and analyze parameter sensitivities. Hence, this point-collocation NIPC method was utilized in this work to obtain the Sobol indices, denoting the sensitivity of each input parameter [40].

In this study, the goal is to analyze the sensitivities of input parameters to AFT, AFMC and AFRH at the working face. According to references [41–43], the flow parameters include ventilation time (*t*), the velocity (*u*), temperature (*T*_*A*_) and relative humidity (*φ*_*A*_) of inflow wind. The geometric parameters include the length (*L*), width (*W*) and height (*H*) of the working face. Additionally, the physical parameters include the thermal conductivity (*λ*) and specific heat capacity (*c*_*ps*_) of coal, the coal output (*G*_*s*_) and the original temperature of the rock (*T*_*n*_). Then the output quantities of interests are the outflow wind temperature (*T*_*b*_), moisture content of outflow wind (*d*_*b*_) and relative humidity of outflow wind (*φ*_*b*_). For sensitivity analyses of input parameters, firstly, the CFD method is employed to calculate the values at sample points generated using the optimal Latin hypercube approach [32, 36, 40]. Subsequently, the point-collocation NIPC method is utilized to calculate the contributions of input parameters to the uncertainty in responses. Finally, sensitivity analyses are conducted to determine the key parameters. Section 2 describes Sobol indices for sensitivity analyses calculated by the point-collocation NIPC method. Section 3 presents the computational details. Then, the calculation results are discussed in Section 4. Finally, Section 5 summarizes the main conclusions of this study.

## 2. Sensitivity analysis approach

Sobol indices are utilized to evaluate the uncertainty contributions under changes of input parameters. The parameter with a higher Sobol index contributes more to the response uncertainty. In this study, the input uncertainties are propagated to the output quantities of interests, such as temperature and relative humidity, with the NIPC method.

The uncertainty response *γ* can be regarded as the series expansion composed of separable stochastic and deterministic terms, based on the point-collocation NIPC theory.

$$\gamma (x,\xi )=\sum_{i=0}^{\infty }\alpha_{i}(x)\psi_{i}(\xi )\approx \sum_{i=0}^{P}\alpha_{i}(x)\psi_{i}(\xi )$$ (1)

where *ψ*_*i*_(*ξ*) and *α*_*i*_(*x*) are orthogonal polynomial basis functions and the deterministic term corresponding to the *i*th mode, respectively. For polynomial chaos expansions, the series is theoretically infinite, but it is truncated in practice. The discrete sum is taken over several output modes [38], and the total number of sample points is as follows [36]

$$N_{s}=n_{p}\cdot (P+1)=n_{p}\cdot \left(\frac{(n+p)!}{n!p!}\right)$$ (2)

where *N*_*s*_ is the total number of samples. *n* is the number of input parameters. *n*_*p*_ is the oversampling ratio. In this work, *n*_*p*_ = 2.0 is assumed for the calculation since it provides a better statistical approximation at each polynomial degree according to the research [36, 40]. *p* is the polynomial order. *p* = 2 is sufficient to capture the heat flux uncertainty, as shown in reference [32].

Then, the surrogate model is built by replacing the left-hand term of Eq (1) with the values obtained from the CFD simulation at the sample points. A linear system composed of *N*_*s*_ equations is formulated, which is solved for the spectrum mode of random variables based on the least-squares approach [36]. This system is given as follows

$$\left.\left(\begin{array}{c} \gamma \left(x,\xi_{0}\right)\\ \gamma \left(x,\xi_{1}\right)\\ \vdots \\ \gamma \left(x,\xi_{N_{s}-1}\right) \end{array}\right.\right)=\left(\begin{array}{cccc} \psi_{0}(\xi_{0}) & \psi_{1}(\xi_{0}) & \cdots & \psi_{P}(\xi_{0})\\ \psi_{0}(\xi_{1}) & \psi_{1}(\xi_{1}) & \cdots & \psi_{P}(\xi_{1})\\ \vdots & \vdots & \ddots & \vdots \\ \psi_{0}(\xi_{N_{s}-1}) & \psi_{1}(\xi_{N_{s}-1}) & \cdots & \psi_{P}(\xi_{N_{s}-1}) \end{array}\right)\left(\begin{array}{c} \alpha_{0}\\ \alpha_{1}\\ \vdots \\ \alpha_{P} \end{array}\right)$$ (3)

The total variance *σ*^2^ can be expressed by polynomial chaos expansions as follows

$$\sigma^{2}=\sum_{i=1}^{P}\alpha_{i}^{2}(x)\langle \psi_{i}^{2}(\xi )\rangle$$ (4)

As shown in references [36, 40], the total variance can be decomposed as

$$\sigma^{2}=\sum_{i=1}^{i=n}\sigma_{i}^{2}+\sum_{1⩽i<j⩽n}^{i=n-1}\sigma_{i,j}^{2}+\sum_{1⩽i<j<k⩽n}^{i=n-2}\sigma_{i,j,k}^{2}+\cdots +\sigma_{1,2,\cdots ,n}^{2}$$ (5)

where the partial variances $\sigma_{i_{1},\cdots ,i_{s}}^{2}$ are calculated as follows

$$\sigma_{i_{1},\cdots ,i_{s}}^{2}=\alpha_{i_{1},\cdots ,i_{s}}^{2}\langle \psi_{i_{1},\cdots ,i_{s}}^{2}(\xi )\rangle ,\;1\leq i_{1}<\cdots <i_{s}\leq n$$ (6)

Sobol indices, utilized to evaluate the sensitivity of input parameters, are calculated by the contributions in the total uncertainty.


$$S_{i_{1},\ldots ,i_{s}}=\frac{\sigma_{i_{1},\ldots ,i_{s}}^{2}}{\sigma^{2}}$$ (7)

Total effect Sobol indices for the global sensitivity analysis, which contain both mixed and individual contributions from each input parameter, are given by

$$S_{T_{i}}=\sum_{L_{i}}\frac{\sigma_{i_{1},\ldots ,i_{s}}^{2}}{\sigma^{2}},\;L_{i}=\{(i_{1},\ldots ,i_{s}):\exists k,1\leq k\leq s,i_{k}=i\}$$ (8)

## 3. Computational details

### 3.1. Geometry and operating conditions

**Fig 1:**
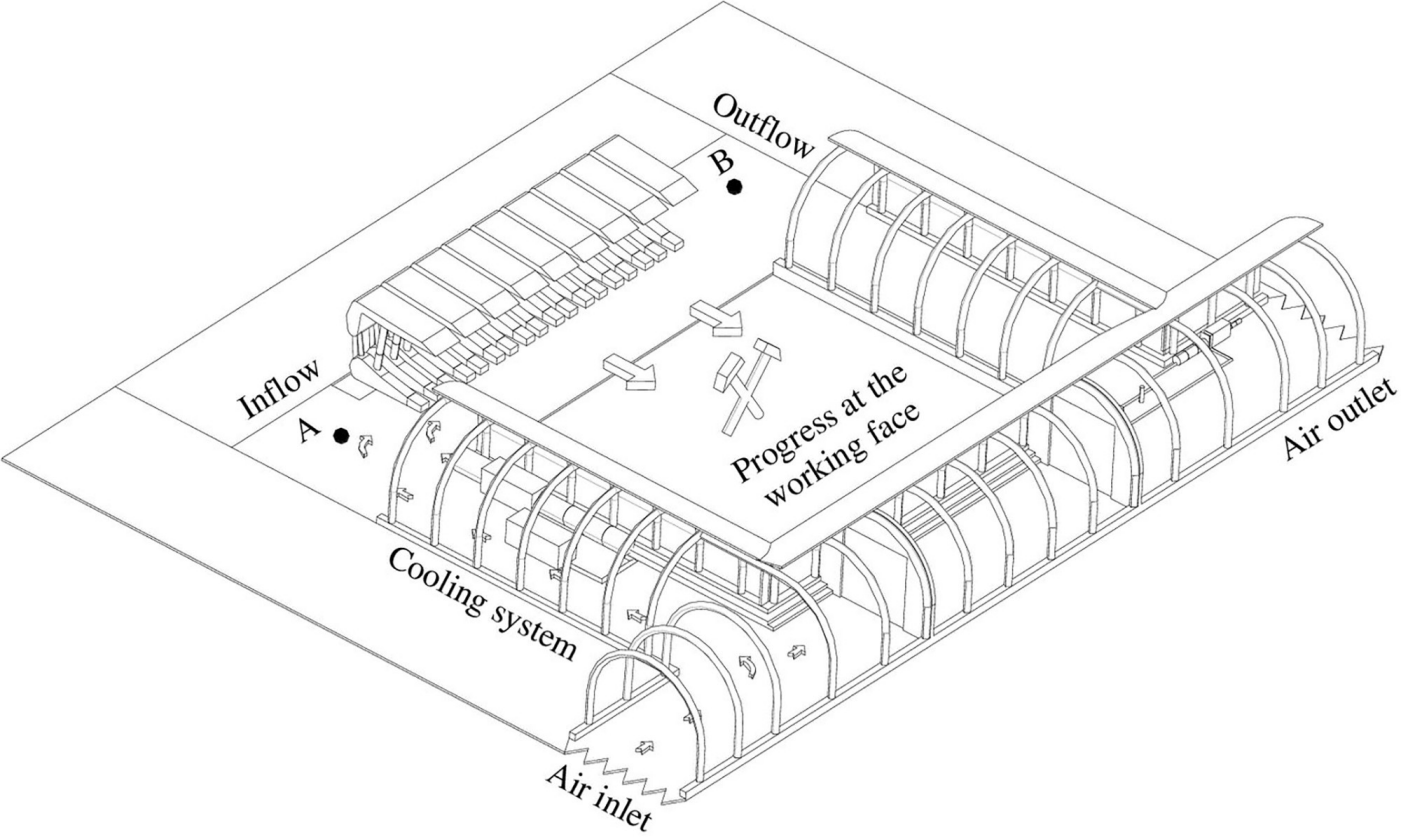
Schematic configuration of U-type ventilation system.

The U-type ventilation system was adopted at the working face [43, 44], as shown in Fig 1. The model size is 230m by 100m. The equivalent diameter and the length of the working face are 3.9m and 180m, respectively. The equivalent diameter and the length of the tunnel are 3.4m and 60m, respectively. The cooling system is 10m away from the working face, and the outlet diameter of the cooling system is 0.85m. The model was built with Solidworks, and the grid was divided with ICEM.

The VOF model employed in Fluent software is adopted here to calculate multiphase flow. Heat and mass transfer between the surrounding rock and airflow can be calculated by the species transport equation and source term macros [45, 46]. The liquid-vapor mass transfer is governed by the vapor transport equation of the Lee model. The relaxation time in the Lee model is 0.5. The quantitative relationship between the temperature and saturated vapor pressure satisfies the Marti Mauersberger equation when the air temperature is below 0°C, and the Geely equation when the air temperature is above 0°C [47]. The user-defined functions were coded using the C language, and the source file was compiled at the working face. The physical parameters of solid, liquid and gas phases are given in Table 1. The operating conditions of numerical simulations are presented in Table 2.

**Table 1:** Physical parameters of each component.

Components Unit	Thermal conductivity W/(m·K)	Density kg/m^3^	Specific heat capacity J/(kg·K)	Thermal diffusivity 10^−6^·m^2^/s	Viscosity N·s/m^2^
Coal	0.32	1317.61	1327.216	0.183	-
Rock	3.37	2545	900.162	1.471	-
Backfill material	0.376	1550	940	0.258	-
Water	0.60	998.2	4182	-	0.001003
Air	0.0242	1.225	1006.43	-	0.000017894

**Table 2:** Operating conditions of numerical simulations.

Operating conditions	Parameter setting	Operating conditions	Parameter setting
Boundary temperature	41.5°C	Relative humidity of inflow wind	*φ*_*A*_ = 94.5%
Initial temperature	41.5°C	Moisture content of inflow wind	*d*_*A*_ = 20.6g/kg (approx.)
Mass fraction of oxygen	0.233	Heat dissipation of local heat sources	Σ*Q*_*i*_ = 244.76kW
Wind velocity of working face	*u* = 1.30m/s	Time step size	*t* = 0.01s
Inflow wind temperature	*T*_*A*_ = 26.4°C	Max iterations	*n* = 20

### 3.2. Sensitivity analysis details

In this study, the sensitivity analysis approach is employed to evaluate the influence of input parameters on AFT, AFMC and AFRH. According to references [41–43], eleven input parameters are treated as aleatory uncertainty variables. These include ventilation time (*t*), the velocity (*u*), temperature (*T*_*A*_) and relative humidity (*φ*_*A*_) of inflow wind, the length (*L*), width (*W*) and height (*H*) of the working face, the thermal conductivity (*λ*) and specific heat capacity (*c*_*ps*_) of coal, the coal output (*G*_*s*_) and the original temperature of the rock (*T*_*n*_). Table 3 shows varied ranges of the input parameters for Cases I and II. The parameter ranges in Case I are taken as a reference, while these in Case II are magnified according to the reference ranges.

**Table 3:** Varied ranges of input parameters. Parameter ranges in Case I are taken as a reference, while these in Case II are magnified according to the reference ranges.

Parameters	Unit	Case I	Case II	
*t*	h	[1, 25]	× 1	-
*u*	m/s	[0.8, 1.8]	× 2	[0.8, 2.8]
*T*_*A*_	°C	[24, 26]	× 2	[22, 26]
*φ*_*A*_	%	[92, 94]	× 2	[90, 94]
*L*	m	[40, 180]	× 1	-
*W*	m	[3, 5]	× 1	-
*H*	m	[3, 5]	× 1	-
*λ*	W/(m·K)	[0.2, 0.6]	× 1	-
*c*_*ps*_	J/(kg·K)	[1100, 1400]	× 1	-
*G*_*s*_	kg/s	[20, 25]	× 1	-
*T*_*n*_	°C	[38, 42]	× 1	-

### 3.3. Code validation and model selection

**Fig 2:**
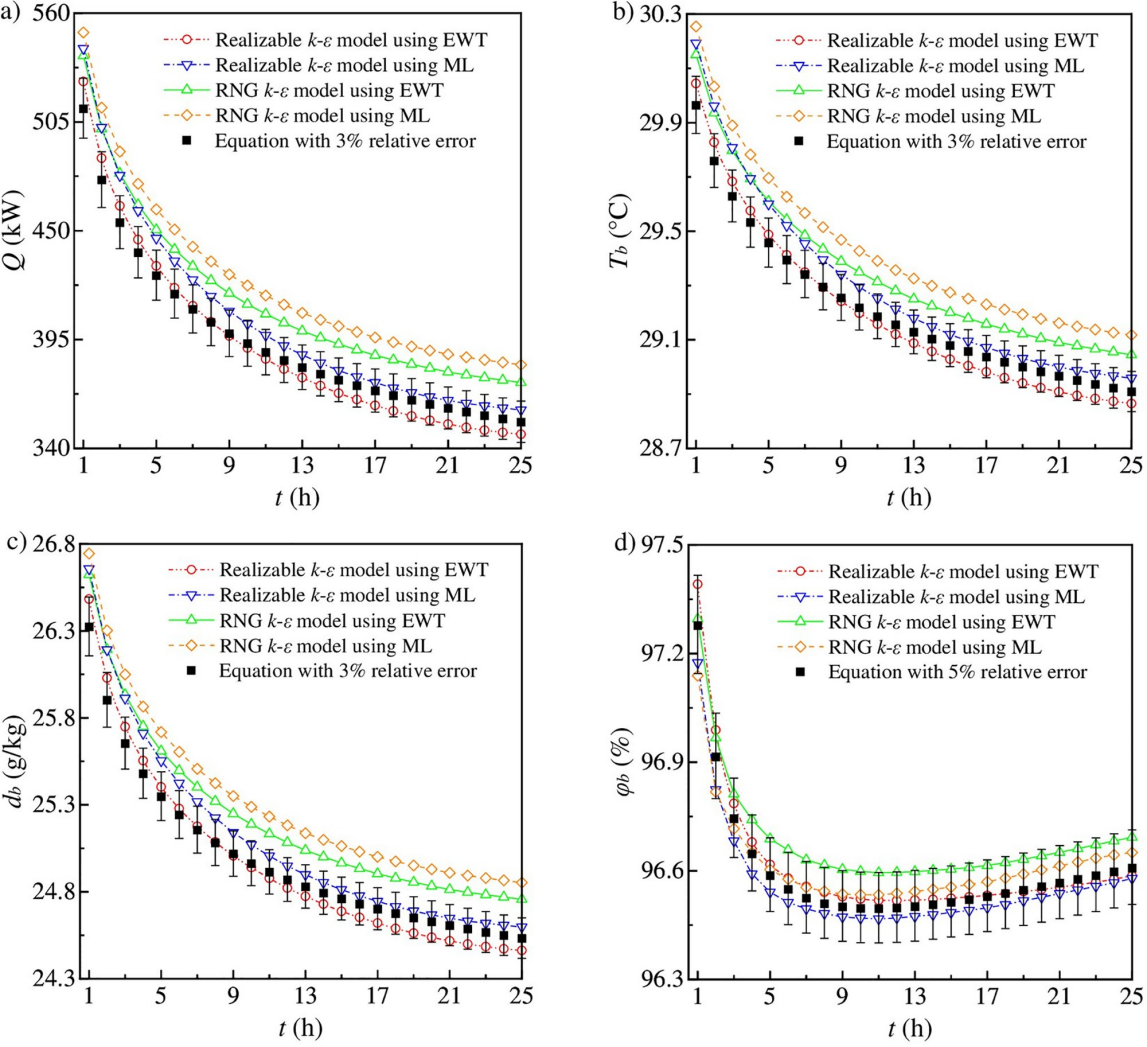
Comparison of predicted equations and numerical simulations. (a) Cooling load of working face. (b) Airflow temperature. (c) Moisture content of airflow. (d) Relative humidity of airflow.

The predicted equations for AFT, AFMC and AFRH at the working face are used to validate the numerical method [8, 41, 42]. The predicted equations can be derived based on the analysis method of heat sources. This derivation is included in the S1 Appendix. Meanwhile, turbulent transport equations and near-wall treatments are important in evaluating heat transfer. Thus, two turbulent models, namely the RNG *k*-*ε* model and the realizable *k*-*ε* model, along with two near-wall treatments, namely the enhanced wall treatment (EWT) and the Menter-Lechner (ML) treatment, are utilized in numerical simulations for finding the best option. Fig 2 plots the comparison of the calculated results from predicted equations and numerical simulations.

According to Fig 2(a)–2(c), for the cooling load, airflow temperature (AFT) and moisture content of airflow (AFMC), the simulated results using EWT are lower than those using ML. The simulated results of the realizable *k*-*ε* model are closer to equation results, while the RNG *k*-*ε* model over-predicts equation results. According to Fig 2(d), for relative humidity of airflow (AFRH), the simulated results of both *k*-*ε* models are in the agreement with equation results, and the errors are within 5%.

In order to quantitatively analyze the difference between simulated results and equation results, the normalized parameter RMS_RN_ of root mean square is calculated as follows [48, 49]:

$${\text{RMS}}_{\text{RN}}=100\sqrt{\frac{1}{N}\sum_{i=1}^{N}{\left(\frac{f_{Eq,i}-f_{i}}{Max(f_{Eq})-Min(f_{Eq})}\right)}^{2}}$$ (9)

where RMS_RN_ is the normalized parameter of root mean square, %; *N* is the total number of data points; *f*_*Eq*,*i*_ is the equation result at the point *i*; *f*_*i*_ is the simulated result at the same point *i*; *Max*(*f*_*Eq*_) is the maximum of the equation result; *Min*(*f*_*Eq*_) is the minimum of the equation result.

By comparing the RMS_RN_ values in Table 4, the realizable *k*-*ε* model using EWT performs best in predicting heat and mass transfer. The RMS_RN_ for all output quantities is between 4.26% and 4.47%. However, the simulated results of the realizable *k*-*ε* model using ML have relatively larger deviations from equation results, with RMS_RN_ ranging from 5.41% to 9.53%. In contrast, the simulated results of the RNG *k*-*ε* model exhibit poor consistency with equation results. Moreover, Reynolds-averaged Navier-Stokes simulations [40, 50] and even Large Eddy Simulation [38, 39] could not well predict the heat flux near vortex intersections, while these studies have proved that numerical simulations can reveal heat flux distributions and flow structures from the perspective of the entire flow field. Improved knowledge of the values of turbulence model closure coefficients can reduce the epistemic uncertainty in integrated output quantities of interests for Reynolds-averaged Navier-Stokes simulations [36]. Consequently, the realizable *k*-*ε* model using the enhanced wall treatment is employed for further simulations.

**Table 4:** RMS_RN_ values of selected numerical methods for predicted equations.

RMS_RN_ results [%]	Realizable *k*-*ε* model using EWT	Realizable *k*-*ε* model using ML	RNG *k*-*ε* model using EWT	RNG *k*-*ε* model using ML
Cooling load	4.30	8.31	13.00	19.03
Temperature	4.47	9.53	13.11	20.60
Moisture content	4.26	8.02	12.98	18.66
Relative humidity	4.43	5.41	11.50	6.46

## 4. Results and discussion

### 4.1. Changes of temperature, moisture content and relative humidity of airflow

**Fig 3:**
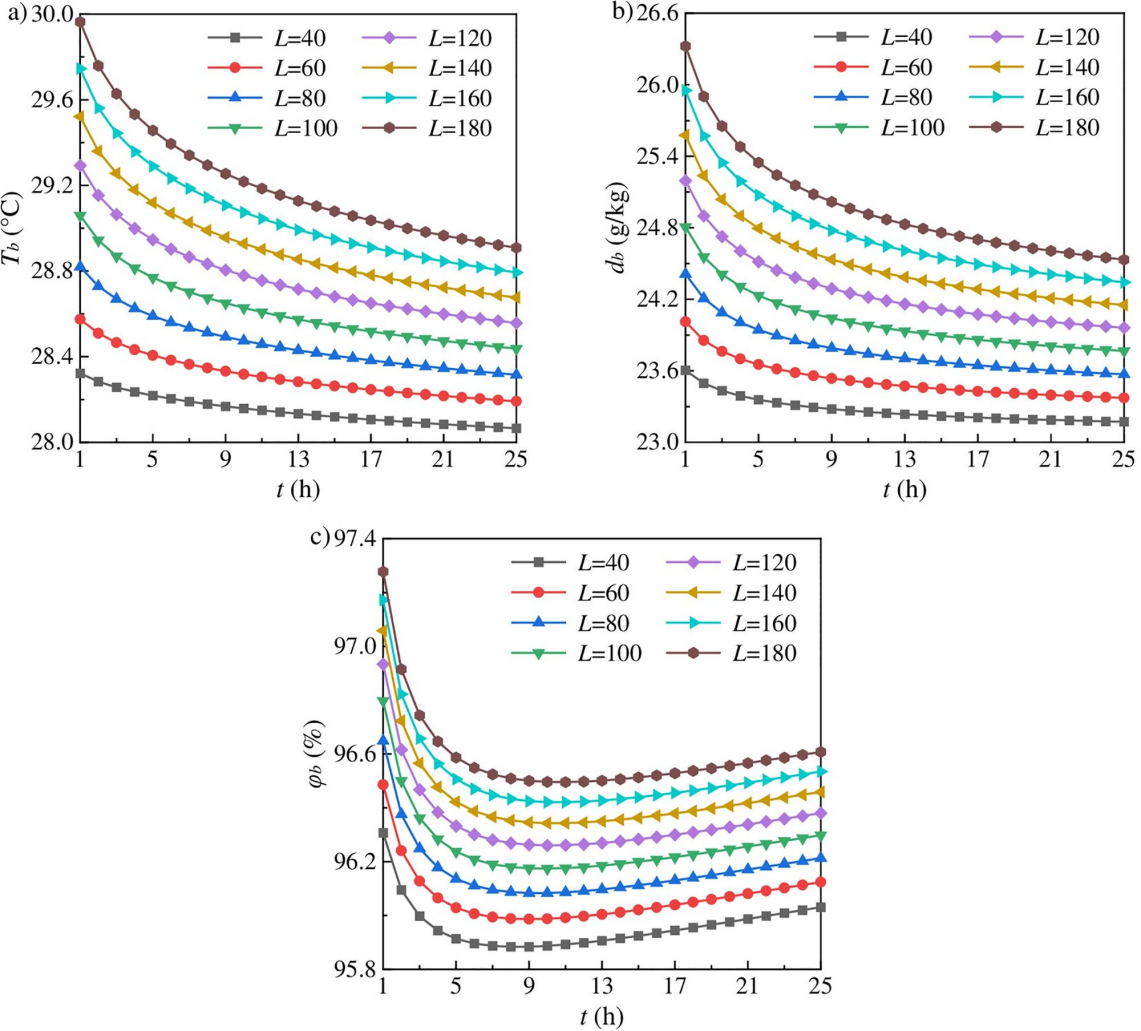
Change curves for (a) airflow temperature, (b) moisture content of airflow, and (c) relative humidity of airflow.

The change curves of AFT, AFMC and AFRH are plotted in Fig 3. As shown in Fig 3(a) and 3(b), AFT and AFMC change through two stages with the increase of ventilation time, i.e., rapid decrease and gradual decrease. Heat and mass transfer between the surrounding rock and airflow involves the complex interactions of heat, moisture and force. The decrease of the wall temperature with increasing ventilation time indicates the decrease of the saturated vapor pressure on the moist wall, and thus the temperature and pressure gradients between the phase interface and airflow decrease. Hence heat dissipation of the surrounding rock, the relative heat source, decreases due to the reduction of the coupled driven force of heat and mass diffusion. This results in the AFT and AFMC decrease with the increase of ventilation time. Moreover, the temperature and vapor pressure of airflow are lower than those of the phase interface, and thus AFT and AFMC gradually increase along the length of the working face.

**Fig 4:**
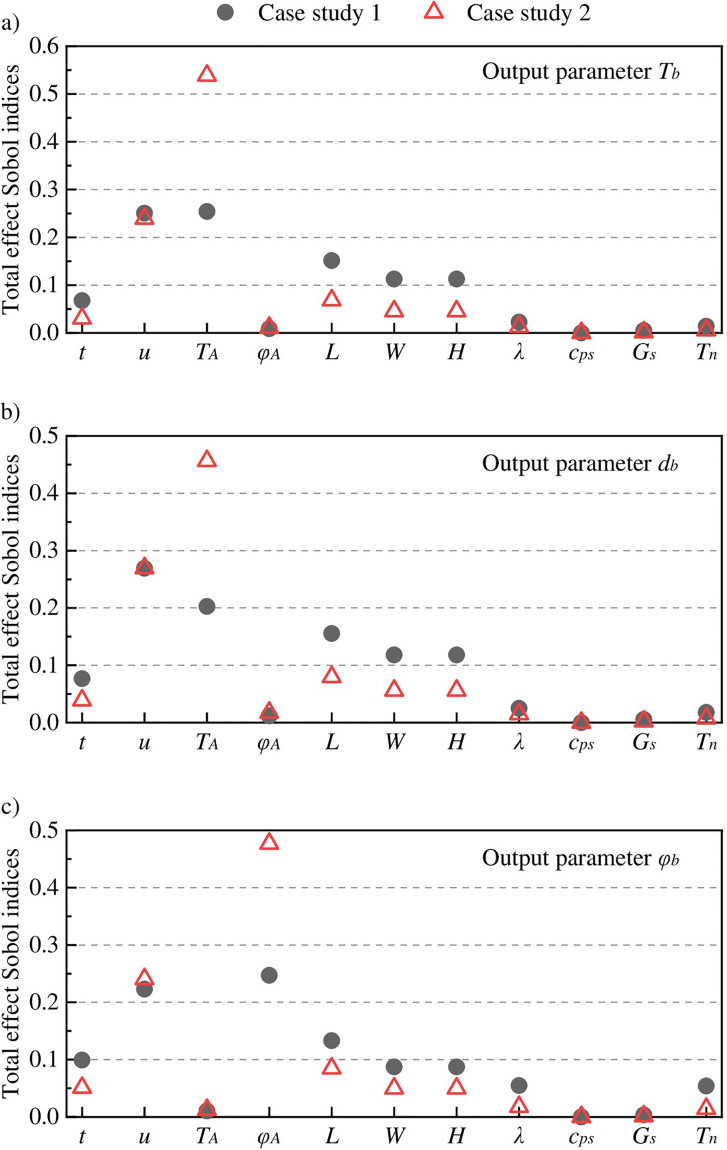
The total effect Sobol indices for (a) airflow temperature, (b) moisture content of airflow, and (c) relative humidity of airflow.

AFRH decreases rapidly and then increases gradually with the increase of ventilation time, as shown in Fig 3(c). AFRH has a minimum value when ventilation time is approximately 10 hours. Firstly, when ventilation time is less than 10 hours, the pressure gradient between the phase interface and airflow decreases rapidly due to the rapid decrease of the wall temperature with increasing ventilation time, and thus resulting in the AFRH decrease, which makes ventilation time *t* have a large contribution to the AFRH uncertainty as presented in Fig 4(c). Secondly, when ventilation time is more than 10 hours, the AFT decrease can result in a rebound change of AFRH, despite both decreases of AFT and AFMC.

### 4.2. Sensitivity analyses of heat and mass transfer

One hundred fifty-six samples of eleven input parameters are allocated using the optimal Latin hypercube approach. The values at these sample points are calculated by CFD simulations, and then utilized to determine the uncertainty contributions of input parameters using the point-collocation NIPC method. Fig 4 reports the total effect Sobol indices of all input parameters in Cases I and II to quantitatively reflect the influence degree on AFT, AFMC and AFRH. Overall, Sobol indices for AFT and AFMC follow a similar distribution, while this index distribution is different from AFRH. The temperature and pressure gradients between the phase interface and airflow are the driven force of heat and mass diffusion. The pressure gradient is almost caused by the difference of the saturated vapor pressure between the phase interface and airflow because the vapor pressure of airflow at the working face is usually close to saturation. Therefore, AFT and AFMC follow a similar index distribution owing to the positive correlation relationship between the temperature and saturated vapor pressure.

Specifically, *T*_*A*_ and *u* contribute more than 0.2 to the uncertainty for AFT and AFMC, see Fig 4(a) and 4(b), making them two main factors that influence heat and mass transfer at the working face. Other significant quantities influencing heat and mass transfer, except *T*_*A*_ and *u*, are the geometric parameters *L*, *W* and *H*. *L*, *W* and *H* have a direct influence on heat dissipation of the surrounding rock, reflecting their relative significance on AFT and AFMC. In addition, the influence degree of ventilation time *t* on AFT and AFMC is relatively small, because the convective heat and mass transfer between the cooling and original airflow is almost completed when the ventilation time is less than 1 hour, which is the fastest stage of the AFT and AFMC changes. The uncertainty contribution of the remaining parameters can be ignored.

Compared to Case I, we observed that the *T*_*A*_ uncertainty contribution in Case II increases significantly while the *u* uncertainty contribution decreases slightly, despite both magnified ranges of *T*_*A*_ and *u*. The reduction of the lower limits for AFT and AFMC in fact is due to the *T*_*A*_ decrease, while the changes of other input parameters including the wind velocity only determine the trend to reach the lower limits for AFT and AFMC. This also indicates that the influence degree of the inflow wind temperature on heat and mass transfer is larger than that of the wind velocity.

Similarly, *φ*_*A*_ and *u* contribute more than 0.2 to the AFRH uncertainty, see Fig 4(c), making them two main factors that influence AFRH. Compared with the AFT and AFMC uncertainty, two other significant quantities influencing AFRH, except *φ*_*A*_, *u*, *L*, *t*, *W* and *H*, are the physical parameters *λ* and *T*_*n*_, while *T*_*A*_ has no remarkable influence on AFRH. The varied *λ* and *T*_*n*_, as well as *t* in Fig 3(c), can produce the diversified wall temperature, and thus the pressure gradient between the phase interface and airflow is altered significantly, which makes *t*, *λ* and *T*_*n*_ have a large influence on AFRH. The uncertainty contribution of the remaining parameters can be ignored. Compared to Case I, we observed that the *φ*_*A*_ uncertainty contribution in Case II increases significantly while the *u* uncertainty contribution increases slightly, despite both magnified ranges of *φ*_*A*_ and *u*. This also indicates that the influence degree of relative humidity of inflow wind on AFRH is larger than that of the wind velocity.

**Fig 5:**
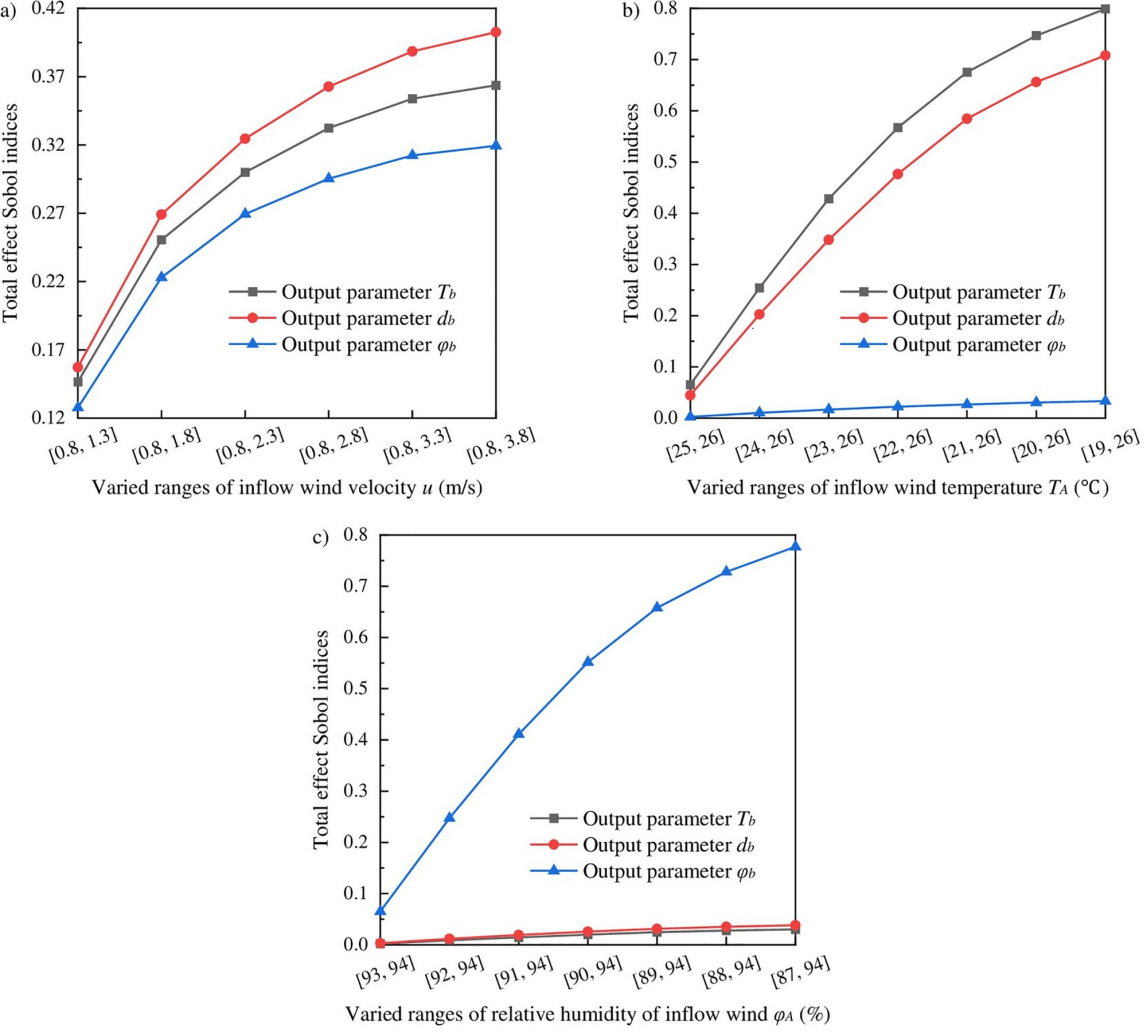
The total effect Sobol indices related to (a) inflow wind velocity *u*, (b) inflow wind temperature *T*_*A*_, and (c) relative humidity of inflow wind *φ*_*A*_. The varied ranges of other input parameters are shown in Case I in Table 3.

When it comes to the sensitivity analyses for single factors, the total effect Sobol indices related to inflow parameters *u*, *T*_*A*_ and *φ*_*A*_ are reported in Fig 5. The varied ranges of other input parameters are assumed the same as Case I in Table 3. As plotted in Fig 5(a), the Sobol indices for *T*_*b*_, *d*_*b*_ and *φ*_*b*_ increase nonlinearly with the increase of the *u* varied ranges, and the changing rate decreases gradually. The Sobol indices for *T*_*b*_, *d*_*b*_ and *φ*_*b*_ nearly reach the upper limit when the wind velocity increases up to 3.8m/s, and this upper limit is between 0.32 and 0.40. In fact, the increase of the wind velocity has little influence on AFT, AFMC and AFRH after the wind velocity above 3.8m/s because the given parameters *T*_*A*_ and *φ*_*A*_ determine the lower limits for AFT, AFMC and AFRH.

With the increase of the *T*_*A*_ varied ranges, the Sobol indices for *T*_*b*_ and *d*_*b*_ increase rapidly while the *φ*_*b*_ Sobol index increases slightly, see Fig 5(b). The Sobol indices for *T*_*b*_ and *d*_*b*_ are above 0.7 when the inflow wind temperature *T*_*A*_ decreases up to 19°C. The varied *T*_*A*_ directly produces diversified temperature and pressure gradients, and thus resulting in various heat and mass diffusion between the phase interface and airflow. Hence the output quantities *T*_*b*_ and *d*_*b*_ are altered dramatically with the inflow wind temperature *T*_*A*_. This finding indicates that *T*_*A*_ is the most sensitive factor that influences heat and mass transfer at the working face, which agrees with the calculated results in Fig 4(a) and 4(b).

Similarly, with the increase of the *φ*_*A*_ varied ranges, the *φ*_*b*_ Sobol index increases rapidly while the Sobol indices for *T*_*b*_ and *d*_*b*_ increase slightly, see Fig 5(c). The *φ*_*b*_ Sobol index is approximately 0.78 when relative humidity of inflow wind *φ*_*A*_ decreases up to 87%. For the given parameter *T*_*A*_, namely the given saturated vapor pressure of inflow wind, the varied *φ*_*A*_ directly produces the diversified pressure gradient between the phase interface and airflow, and thus AFRH is altered dramatically with relative humidity of inflow wind *φ*_*A*_, which indicates that *φ*_*A*_ is the most sensitive factor that influences AFRH, agreeing with the calculated results in Fig 4(c).

Parameter significance is classified by the contribution to the AFT, AFMC and AFRH uncertainty, as listed in Table 5. As shown in this table, the lower temperature and the higher velocity of inflow wind can bring the better cooling effect. The lower relative humidity and the higher velocity of inflow wind can bring the better dehumidification effect.

**Table 5:** Classification of parameter significance.

Parameter significance uncertainty contribution	Main parameters [0.2, 1.0]	Significant parameters [0.05, 0.2)	Insignificant parameters [0, 0.05)
*T*_*b*_ *d*_*b*_	*T*_*A*_ *u*	*L* *W* *H* *t*	*λ* *T*_*n*_ *φ*_*A*_ *G*_*s*_ *c*_*ps*_
*φ*_*b*_	*φ*_*A*_ *u*	*L* *t* *W* *H* *λ* *T*_*n*_	*T*_*A*_ *G*_*s*_ *c*_*ps*_

## 5. Conclusions

In this study, sensitivity analyses of the working face parameters on AFT, AFMC and AFRH are performed to find their main contributors. The variations of flow, geometric and physical conditions are regarded as uncertainty sources. The values at one hundred fifty-six sample points are simulated. Subsequently, the point-collocation NIPC method was utilized to obtain the Sobol indices, denoting the sensitivity of each input parameter. Overall, total effect Sobol indices for AFT and AFMC follow a similar distribution, while this index distribution is different from AFRH. The conclusions are drawn specifically as follows:

(1) AFT and AFMC change through two stages with the increase of ventilation time, i.e., rapid decrease and gradual decrease. AFRH decreases rapidly and then increases gradually with the increase of ventilation time. The minimum AFRH occurs at approximately 10 hours of ventilation time.
(2) *T*_*A*_ and *u* contribute more than 0.2 to the uncertainty for AFT and AFMC in both Cases I and II, making them two top factors influencing heat and mass transfer at the working face. Other significant factors influencing heat and mass transfer include *L*, *W*, *H* and *t*, while the uncertainty contribution of the remaining parameters can be ignored.
(3) *φ*_*A*_ and *u* contribute more than 0.2 to the AFRH uncertainty in both Cases I and II, making them two top factors influencing AFRH. Compared to AFT and AFMC, two other significant factors influencing AFRH, apart from *L*, *t*, *W* and *H*, are the physical parameters *λ* and *T*_*n*_. Notably, *T*_*A*_ does not exhibit remarkable influence on AFRH.
(4) The *u* contribution to the AFT, AFMC and AFRH uncertainty increases nonlinearly as the wind velocity increases from 0.8m/s to 3.8m/s. Upon reaching a wind velocity of 3.8m/s, the *u* uncertainty contribution nearly reaches the upper limit, ranging between 0.32 and 0.40. This finding indicates that the influence of wind velocity on AFT, AFMC and AFRH decreases significantly beyond a wind velocity of 3.8m/s.
(5) The *T*_*A*_ contribution to the AFT and AFMC uncertainty exceeds 0.7 as *T*_*A*_ decreases from 26°C to 19°C, indicating that *T*_*A*_ is the most sensitive factor influencing heat and mass transfer at the working face. Similarly, the *φ*_*A*_ contribution to the AFRH uncertainty is approximately 0.78 as *φ*_*A*_ decreases from 94% to 87%, indicating that *φ*_*A*_ is the most sensitive factor influencing AFRH.

## Supporting information

S1 Appendix Predicted equations of temperature and relative humidity of airflow. (DOC)
