# Supplementary material for: Sensitivity analysis of heat and mass transfer at working face in high-temperature mine
Source: PLoS One. 2024 Jun 28;19(6):e0306269. doi: 10.1371/journal.pone.0306269 (PMC11213306; doi:10.1371/journal.pone.0306269)
Supplement: S1 Appendix — (DOC) [file pone.0306269.s001.doc]

**Appendix.** **Predicted equations of temperature and relative humidity of airflow**

In this appendix, the predicted equations of AFT, AFMC and AFRH are presented in detail. The analysis method of heat sources is capable of calculating the thermal energy released at the working face, including heat dissipation from the surrounding rock and other local heat sources. In the thermal environment analysis, heat dissipation from the surrounding rock is generally calculated as [41]

$Q_{\tau}=K_{\tau}(T_{n}-T_{f})UL$ (A1)

where *Q_τ_* is heat dissipation from the surrounding rock, kW; *T_n_* is the original temperature of the rock, K; *T_f_* is the airflow temperature, K; *U* is the tunnel cross-section perimeter, m; and *L* is the tunnel length, m. The unsteady heat transfer coefficient *K_τ_* and the convective heat transfer coefficient *h* can be expressed as [41]

(A2)

$h=2.728\varepsilon u^{0.8}$ (A3)

where *λ* is the thermal conductivity of the rock, W/(m·K); *Fo* is the Fourier number; *a* is the thermal diffusivity of the rock, m^2^/s; *t* is ventilation time, s; *ε* is the roughness coefficient of the rock surface; and *u* is the wind velocity, m/s.

Additionally, Table A1 lists the local heat sources at the working face, excepting heat dissipation of the surrounding rock [8,41]. The measured parameters of the working face, as provided in references [43,44], are given in Table A2 for calculating the heat dissipation of local heat sources. The calculated result is Σ*Q_i_* = 244.76kW.

**Table A1. Local heat sources at the working face.**

| Local heat sources | Calculation equation | Local heat sources | Calculation equation |
| --- | --- | --- | --- |
| Coal oxidation | *Q*_0_ = *q*_0_*ULu*^0.8^ | Ore transportation | *Q_k_* = 0.7*G_s_c_ps_*Δ*t* |
| Equipment | *Q_l_* = 0.1*N* | Human body | *Q_R_* = 0.7*q_R_n* |
| Ore cooling | *Q_c_* = 37.2*G_c_* | Heat dissipation of local heat sources | Σ*Q_i_* = *Q*_0_ + *Q_l_* + *Q_c_* + *Q_k_* + *Q_R_* |

where *q*_0_ is heat dissipation of coal oxidation per unit area, kW/m^2^. *q*_0_ can be treated as a constant since the wall commonly consists of newly exposed coal during the progress of the working face. *N* is the total power of equipment, kW; *G_c_* is the round-the-clock coal output at the working face, t/d; *G_s_* is the coal output, kg/s; *c_ps_* is the specific heat capacity of coal, J/(kg·K); Δ*t* is the decreased temperature during ore transportation, K; *q_R_* is the metabolic rate, W/person; and *n* is the number of workers at the working face.

**Table A2. Measured parameters of the working face.**

| Measured parameters | Value | Measured parameters | Value |
| --- | --- | --- | --- |
| Round-the-clock coal output | *G_c_*=1500t/d | Decreased temperature during ore transportation | Δ*t*=0.815°C |
| Total power of equipment | *N*=1335kW | Heat dissipation of coal oxidation | *q*_0_=17W/m^2^ |
| Latent heat of water evaporation | *γ*=2501kJ/kg | Length of the working face | *L*=180m |
| Atmospheric pressure | *B*=101325Pa | Roughness coefficient of the rock surface | *ε*=3 |
| Lewis number | *Le*=0.892 | Cross-section perimeter of the working face | *U*=18.4m |
| Number of workers | *n*=15person | Cross-section area of the working face | *F*=18m^2^ |
| Metabolic rate | *q_R_*=470W/person | Equivalent diameter of the working face | *D*=3.9m (approx.) |

The thermal energy released from heat sources of the working face can increase both the temperature and moisture content of airflow due to plenty of water in the tunnel. The latent-sensible heat ratio is derived based on differential equations of heat and mass transfer [42].

(A4)

where *ζ* is the coefficient of the latent-sensible heat ratio; *Q_q_* is latent heat transfer per unit area, W/m^2^; *Q_x_* is sensible heat transfer per unit area, W/m^2^; *γ* is latent heat of water evaporation, kJ/kg; *c_p_* is the specific heat capacity of air at the constant pressure, J/(kg·K); *Le* is the Lewis number; *B* is the atmospheric pressure, Pa; *φ* is relative humidity of air, %; *p_fb_* is the saturated vapor pressure of air, Pa; *p_wb_* is the saturated vapor pressure of the moist rock wall, Pa; and *T_w_* is the wall temperature, K.

Then, the amounts of sensible heat and latent heat between the surrounding rock and airflow can be obtained according to the latent-sensible heat ratio [8,42].

(A5)

(A6)

where *Q_Q_* and *Q_X_* are the amounts of sensible heat and latent heat, respectively. *ϕ* is the moist wall coefficient and is set to 0.8. Generally, the moist wall coefficient is not directly measured but empirically obtained by the best fitting of measured data [42].

Fig A.6 illustrates the schematic diagram of the U-type ventilation system at the working face. The predicted equations of AFT and AFMC at the working face are derived based on the first law of thermodynamics [42].

(A7)

(A8)

where *T* is the air temperature, K; *d* is moisture content of air, kg/kg; *ρ* is the air density, kg/m^3^; *F* is the tunnel cross-section area, m^2^.


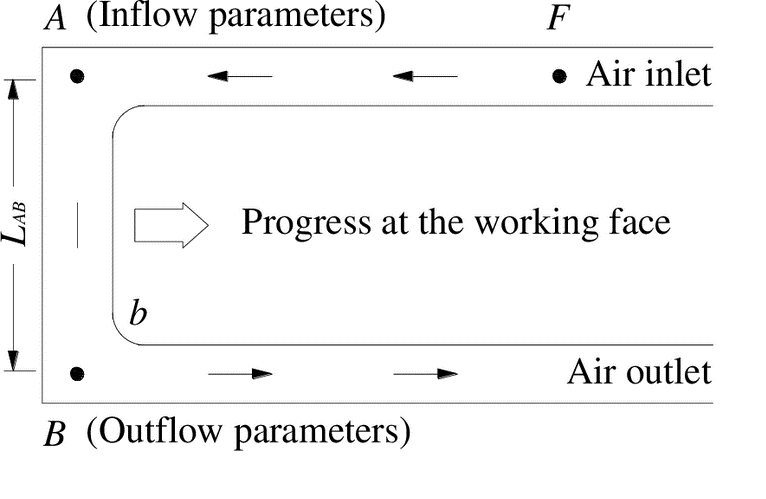


**Fig A.6. Schematic diagram of U-type ventilation system.**

Relative humidity of airflow can be calculated according to the relationship between the vapor pressure and moisture content [43].

$\phi_{B}=\frac{1}{p_{fb,B}}\cdot\frac{d_{B}B}{d_{B}+0.622}$ (A9)

The air heating and cooling during mine ventilation occur at normal atmospheric pressure. The cooling load of the working face can be calculated using the enthalpy difference method [43].

$Q=\rho Fu(i_{B}-i_{A})$ (A10)

where *Q* is the cooling load of the working face, kW. The air enthalpy *i* can be expressed as

$i=Tc_{p}+d\gamma$ (A11)
